# Use of Intravenous Anakinra for Management of Pediatric Cytokine Storm Syndromes at an Academic Medical Center

**DOI:** 10.1177/00185787221142470

**Published:** 2023-02-14

**Authors:** Lirong Yang, Sandra Lowry, Travis Heath

**Affiliations:** 1 UNC-Chapel Hill, Chapel Hill, NC, USA; 2 Duke University Hospital, Durham, NC, USA

**Keywords:** MIS-C, COVID-19, anakinra, pediatrics, off-label use

## Abstract

**Background:** Off-label intravenous (IV) route of anakinra is increasingly recognized to enable higher and faster maximal plasma concentrations than subcutaneous route for treatment of cytokine storm syndromes. **Objective:** To describe off-label indications of IV anakinra, corresponding dosing and safety profiles, particularly during the coronavirus disease 19 (COVID-19) pandemic. **Methods:** A retrospective, single-cohort study was conducted at an academic medical center to evaluate use of IV anakinra in hospitalized pediatric patients (age ≤21 years). Institutional Review Board review was considered exempt. The primary endpoint was the primary indication(s) for IV anakinra. The key secondary endpoints were dosing of IV anakinra, previous immunomodulatory therapies, and adverse events. **Results:** Of 14 pediatric patients, 8 (57.1%) received IV anakinra for treatment of multisystem inflammatory syndrome in children (MIS-C) associated with COVID-19, whereas 3 and 2 were treated for hemophagocytic lymphohistiocytosis (HLH) and flares of systemic onset juvenile idiopathic arthritis (SoJIA), respectively. The initial dosing regimen of IV anakinra for MIS-C associated with COVID-19 was a median dose of 2.25 mg/kg/dose with a median dosing interval of 12 hours for a median initial treatment duration of 3.5 days. Eleven (78.6%) patients received previous immunomodulatory therapies (IV immune globulin [n = 10; 71.4%] and steroids [n = 9; 64.3%]). No adverse drug events were documented. **Conclusion:** IV anakinra was used off-label for treatment of MIS-C associated with COVID-19, HLH and SoJIA flares in critically ill patients with no adverse drug events documented. This study helped ascertain the off-label indications of IV anakinra and corresponding patient characteristics.

## Introduction

Cytokine storm syndromes are life-threatening conditions characterized by fulminant hypercytokinemia that can be triggered by conditions such as sepsis, autoimmune disorders, hemophagocytic lymphohistiocytosis (HLH), chimeric antigen receptor T-cell (CAR-T) therapies, and, more recently, coronavirus disease 2019 (COVID-19).^1,2^ Multisystem inflammatory syndrome in children (MIS-C) is a rare critical condition (estimated to affect 5.1 persons per 1 000 000 person-months for COVID-19 infections reported in the US from April to June 2020) among persons younger than 21 years.^3^ This emerging cytokine storm syndrome is characterized by fever, laboratory evidence of inflammation and multi-organ dysfunction that manifests later in the course of COVID-19 infection in patients less than or equal to 21 years of age. Anakinra, an interleukin-1 receptor antagonist, has been reported as a treatment strategy for MIS-C.^4,5^ At the time of this article’s publication, anakinra is FDA-approved for subcutaneous administration for treatment of rheumatoid arthritis, neonatal-onset multisystem inflammatory disease, and deficiency of interleukin-1 receptor antagonist.^6^ Subcutaneous (SubQ) anakinra is also often used for off-label indications, including treatment of familial Mediterranean fever, juvenile idiopathic arthritis, acute gout flares, and recurrent pericarditis, along with its use for the cytokine storm syndromes listed previously.^7^

In critically ill patients with cytokine storm syndromes, SubQ anakinra could potentially result in unreliable drug absorption, requiring multiple painful injections to achieve high doses, and might be contraindicated in patients with thrombocytopenia and coagulopathy.^2^ Intravenous (IV) anakinra has a shorter half-life, higher maximum concentration, and similar AUC compared with SubQ anakinra.^2^ Therefore, IV anakinra, an off-label route, is considered in clinically unstable patients where a rapid onset with higher and more consistent maximal plasma concentration is required and/or when patients have subcutaneous edema, severe thrombocytopenia, or neurological involvement.^2^

Several studies have reported these off-label uses of anakinra, including use of the off-label IV route of administration, with a compelling benefit-to-risk profile in patients with secondary HLH or macrophage activation syndrome (sHLH/MAS).^2,8-15^ As noted in these studies, anakinra IV or SubQ therapy should be started within 12 hours of sufficient clinical suspicion of cytokine storm with a starting dose of ≥1 to 2 mg/kg/day (max 8 mg/kg/day).^2^ In the context of MIS-C, high-dose anakinra (>4 mg/kg/day IV or SubQ and often up to 5-10 mg/kg/day) has been suggested as an immunomodulatory therapy in patients refractory to glucocorticoid or intravenous immune globulin (IVIG) or in patients with contraindications to long-term use of glucocorticoids.^5^ Dose adjustment may be required at CrCl <30 mL/min/1.73 m^2^ since anakinra is renally eliminated.^6^

Although IV anakinra is increasingly recognized as an important treatment modality for a variety of cytokine storm syndromes, the true frequency of IV anakinra use in current clinical practice is unknown.^2^ This retrospective cohort study was designed to ascertain the off-label uses of IV anakinra and corresponding patient characteristics in an academic medical center setting, particularly during the COVID-19 pandemic where cases of MIS-C may have occurred. The objective of this study was to provide additional evidence for off-label indications of IV anakinra, including corresponding dosing strategies and relevant safety information.

## Methods

This retrospective, single-center cohort study was considered exempt from full Institutional Review Board (IRB) review as patient consent was not required due to the retrospective nature of this study. Hospitalized patients aged 21 years or younger and admitted to the academic medical center for cytokine storm syndromes from January 1, 2020 to June 30, 2021 were included in this study. At our institution, patients who met these criteria were diagnosed with MIS-C associated with COVID-19: age <21 years; fever >24 hours; lab evidence of inflammation (leukocytosis/CRP); evidence of clinically severe illness requiring hospitalization with at least 2 organ system involvement; positive SARS-CoV2 PCR or serology or COVID-19 exposure within 4 weeks prior to admission; and without alternative diagnosis. Patient demographic information was collected, including age, gender, and race. Clinical characteristics of patients at baseline were reviewed, including total body weight, body mass index (BMI), underlying health conditions, organ-system involvement, serum creatinine (SCr), and inflammatory biomarkers (C-reactive protein [CRP], ferritin, and D-dimer). The organ-system involvement was categorized as involvement of cardiovascular, respiratory, renal, neurologic, gastrointestinal, hematologic, mucocutaneous, and musculoskeletal system.^16^ The primary endpoint was the primary indication(s) for use of IV anakinra. The 30-day survival rate was recorded for informational purpose only. The secondary endpoints were immunomodulatory therapies used prior to IV anakinra, prescribed dosing regimens of IV anakinra (dose, frequency, and treatment duration), presence of concomitant infections, change in renal function from the baseline (before initiation of IV anakinra) to the last dose of IV anakinra, change in inflammatory markers (including CRP, ferritin, and D-dimer) from the baseline (before initiation of IV anakinra) to the last dose of IV anakinra, initial time to fever resolution (defined as an afebrile state for at least 24 hours) after initiation of IV anakinra, documented adverse events attributed to use of IV anakinra, and the number of patients who transitioned to a maintenance SubQ anakinra regimen. Data were extracted from the electronic medical record (EMR) using SAP Business Objects MUE Universe software (Newtown Square, PA). Descriptive statistics (medians, percentages, and interquartile ranges) were used to summarize findings. The stability for IV anakinra preparations has not been well established through studies. At our institution, IV anakinra was prepared by diluting the ordered dose in 0.9% sodium chloride as an intravenous piggyback (IVPB) with a final concentration between 1 and 4 mg/mL. The infusion bag is protected from light with a beyond use date of 8 hours.^17^ The infusion is typically administered over 30 to 60 minutes.

## Results

### Patient Population

A total of 14 patients who received IV anakinra therapy for treatment of cytokine storm syndromes were included for analysis, 10 (71.4%) of whom were treated in the pediatric intensive care unit (PICU). Demographic information and clinical characteristics of study patients can be found in Table 1. Most (11 [78.6%]) patients had evidence of at least 1 organ-system involvement. The most commonly involved organ systems were cardiovascular (9 [64.3%]) and respiratory (3 [21.4%]). Three patients had no organ-system involvement. Three patients had acute kidney injury (AKI) but all 3 had resolution of AKI prior to initiation of IV anakinra. The median number of underlying conditions for each patient was 1, with the most common underlying clinical conditions being congenital heart disease in 4 patients and anemia in 4 patients followed by obesity in 3 patients.

**Table 1.** Demographic and Clinical Characteristics of Study Patients at Baseline.

Characteristics	Patients on IV anakinra (n = 14)
Age—y	6 (4.0-10.5)
Age group	
<1 y	1 (7.1)
1-4 y	4 (28.6)
5-9 y	5 (35.7)
10-14 y	2 (14.3)
15-21 y	2 (14.3)
Female sex	8 (57.1)
Race	
White	4 (28.6)
Black	6 (42.8)
Other	4 (28.6)
Total body weight— kg	26.5 (14.8-37.4)
BMI—kg/m^2^	19.6 (15.0-25.0)
Previously healthy	4 (28.6)
Underlying conditions per patient—n	1 (0.3-3.0)
Organ-system involvement per patient—n	1 (1.0-2.0)
PICU admission	10 (71.4)
Baseline serum creatinine—mg/dL	0.6 (0.4-0.9)
Baseline CRP—mg/dL	7.6 (3.4-21.2)
Baseline ferritin—ng/mL	731 (555.3-2140.3)
Baseline D-dimer—ng/mL FEU	13 606 (3398.0-20 335.0)

*Note*. Data are presented as n (%) or median (IQR). BMI = body mass index; CRP = C-reactive protein; FEU = fibrinogen equivalent units; IQR = interquartile range; PICU = pediatric intensive care unit.

### Primary Endpoint

A majority (8 [57.1%]) of the patients received IV anakinra for treatment of MIS-C associated with COVID-19 infection, whereas 3 (21.4%) patients were treated for HLH and 2 (14.3%) patients were treated for flares of systemic onset juvenile idiopathic arthritis (SoJIA). One patient was treated for possible cytokine storm/hyperinflammatory state (see Table 2).

**Table 2.** Primary Indications for Use of Intravenous Anakinra and 30-Day Survival Rate.

Primary indication	N (%) of patients	Survival rate—N (%)
MIS-C associated with COVID-19 infection	8 (57.1)	8 (100.0)
HLH	3 (21.4)	1 (33.3)
Systemic onset juvenile idiopathic arthritis flare	2 (14.3)	2 (100.0)
Possible cytokine storm/hyperinflammatory state	1 (7.1)	0

*Note*. COVID-19 = coronavirus disease 19; HLH = hemophagocytic lymphohistiocytosis; MIS-C = multisystem inflammatory syndrome in children.

### Secondary Endpoints

The initial dosing regimen of IV anakinra for treatment of MIS-C associated with COVID-19 was a median dose of 2.3 mg/kg with a median dosing interval of 12 hours for a median initial treatment duration of 3.5 days (see Table 3). The dose was tapered in 4 (28.6%) patients including a 50% dose reduction in 2 patients and a 50% increase in dosing interval in 1 patient (see Supplemental Appendix). Three (21.4%) patients required dose escalation after initiating IV anakinra due to either deterioration of clinical status (2 patients with more frequent dosing) or no response (1 patient with a 50% increase in dose) (see Supplemental Appendix). The patient with no response to IV anakinra was later diagnosed with refractory MIS-C. All patients (n = 8) with MIS-C were able to be tapered down and eventually discontinue IV anakinra with a median total treatment duration of 8 days (see Table 3).

**Table 3.** Intravenous (IV) Anakinra Dosing Regimen for Primary Indications.

Primary indications	Initial dose—mg/kg/dose	Initial IV dosing interval—hours	Initial treatment duration^a^—days	Total treatment duration—days	Dose range^d^—mg/kg/dose
MIS-C associated with COVID-19 infection	2.3 (2.0-4.0)	12.0 (12.0-12.0)	3.5 (2.0-5.0)	8.0 (5.8-10.8)	1.0-5.0
HLH	5.0 (3.1-7.5)	24.0 (15.0-24.0)	N/A^b^	11.0 (6.0-11.8)	1.3-10.0
SoJIA flares	3.3 (2.9-3.6)	15.0 (10.5-19.5)	4.5 (3.8-5.3)	6.5 (6.3-6.8)	2.5-4.0
Possible cytokine storm/hyperinflammatory state^c^	2.0	12.0	2.0	2.0	2.0

*Note*. Data are presented as median (IQR). COVID-19 = coronavirus disease 19; HLH = hemophagocytic lymphohistiocytosis; MIS-C = multisystem inflammatory syndrome in children; SoJIA = systemic onset juvenile idiopathic arthritis.

a The initial treatment duration is defined as the duration when IV anakinra was originally ordered as initial dosing regimen.

b The dose and dosing interval were not changed for the indication of HLH, so the initial treatment duration was not applicable.

c Only 1 patient had possible cytokine storm/hyperinflammatory state, so the median (IQR) was not calculated.

d Dose range is defined as the minimum to maximum dose given during the total treatment duration.

The dosing regimens used off-label for treatment of HLH, SoJIA, and possible cytokine storm/hyperinflammatory state were highly variable among patients (see Table 3). The median overall treatment duration for all 4 primary indications included 14 patients was 7 days. The majority (11 [78.6%]) of patients had received previous immunomodulatory therapies (see Table 4), which included 10 (71.4%) patients that received IVIG and 9 (64.3%) patients that received glucocorticoids (methylprednisolone, prednisone, and/or hydrocortisone). Eight (57.1%) patients received both IVIG and glucocorticoid therapies before receiving IV anakinra. Immunomodulatory therapies other than IVIG and glucocorticoid (tocilizumab, methotrexate, tacrolimus, canakinumab, and rilonacept) were all given in 1 patient with frequent and persistent SoJIA flares prior to IV anakinra.

**Table 4.** Secondary Endpoints of Intravenous Anakinra.

Secondary endpoints	Patients on IV anakinra (n = 14)
Previous immunomodulatory therapies	11 (78.6)
IVIG	10 (71.4)
Steroids	9 (64.3)
Other^a^	1 (7.1)
Concomitant infections	11 (78.6)
COVID-19	8 (57.1)
Candidemia	2 (14.3)
EBV viremia	1 (7.1)
Bacterial infection	0
Reduction in creatinine—mg/dL	0.2 (0.3-0)
Reduction in CRP—mg/dL	11.0 (22.3-0.8)
Reduction in ferritin—ng/mL	362.0 (1369.3-158.0)
Reduction in D-dimer—ng/mL FEU	8717.5 (13 164.5-1036.5)
Drug related adverse events^b^	0
Initial time to fever resolution—h	17.0 (1.2-37.8)
Transition to maintenance subcutaneous anakinra	2 (14.3)

*Note*. Data are presented as n (%) or median (IQR). COVID-19 = coronavirus disease 19; CRP = C-reactive protein; EBV = Epstein–Barr virus; FEU = fibrinogen equivalent units; IQR = interquartile range; IVIG = intravenous immune globulin.

a Other immunomodulatory therapies were tocilizumab, methotrexate, tacrolimus, canakinumab, and rilonacept, used in the same patient.

b None was reported for these patient admissions.

The majority (11 [78.6%]) of patients had 1 concomitant infection (see Table 4), including COVID-19 (8 [57.1%]), candidemia (2 [14.3%]), and Epstein–Barr viremia [EBV] (1 [7.1%]). No patients developed bacterial infections, and no patients developed more than 1 infection. Of the 14 patients receiving IV anakinra, there were median reductions of serum creatinine by 0.2 mg/dL, of CRP by 11.0 mg/dL, of ferritin by 362.0 ng/mL, and of D-dimer by 8717.5 ng/mL, from the baseline to the last dose of IV anakinra. One patient with SoJIA flares experienced elevations in CRP, ferritin, and D-dimer during the treatment. One patient with MIS-C experienced an elevation in CRP by 1.85 mg/dL at the end of the treatment. All other patients had reductions in CRP, ferritin, and D-dimer. Median time to fever resolution after IV anakinra administration was 17 hours.

Three (21.4%) patients died before completing treatment, including 2 patients with HLH and 1 patient with possible cytokine storm/hyperinflammatory state. All deaths were attributable to the underlying cardiovascular diseases. The 2 patients with HLH received high dose IV anakinra (>4 mg/kg/day) for more than 10 days with the direct death cause being multiorgan failure. No adverse drug events were documented except for tachyphylaxis which developed in 1patient with a SoJIA flare on SubQ anakinra prior to admission. Two (14.3%) patients (1 patient with MIS-C and 1 patient with SoJIA) were transitioned to a SubQ anakinra regimen when deemed clinically stable.

## Discussion

In this study, IV anakinra therapy was used off-label for treatment of MIS-C associated with COVID-19, HLH, and management of SoJIA flares in critically ill pediatric patients including those with previous glucocorticoid or IVIG treatment. Patients treated with IV anakinra maintained baseline renal function and experienced a reduction in inflammatory biomarkers, including CRP, ferritin, and D-dimer.

These 3 off-label indications of IV anakinra have been previously reported.^2,5^ Because MIS-C associated with COVID-19 is an emerging and rare pediatric syndrome, treatment strategies have been largely extrapolated from data in adults or data from other pediatric conditions with similar clinical features.^3,5,16^ Although the American College of Rheumatology Clinical Guidance for Pediatric Patients with MIS-C Associated with COVID-19 recommends high-dose IV or SubQ anakinra at a dose of > 4 mg/kg/day, only half of the patients with MIS-C in this study started with IV anakinra at a dose of 4 mg/kg/day. The other half were started at lower or higher dosing.^5^ The maximum dose of IV anakinra in this study for treatment of MIS-C was 16 mg/kg/day, given to a 3-year-old female patient who received hydrocortisone and IVIG previously without adequate response. This dosing regimen was consistent with the doses reported for HLH/MAS with critical illness in a retrospective case series (2-4 mg/kg/dose IV or SubQ every 6-24 hours), though the optimal dosing regimens for MIS-C associated with COVID-19 have not yet been established.^11^ In a retrospective descriptive study, anakinra at a dose of 4 to 10 mg/kg/day (median, 6.6) for a median treatment duration of 11 days was administered in 23 patients with MIS-C, and complete remission was achieved in 86.9% of these patients.^18^

IV anakinra is considered a first-line treatment option for hemophagocytic lymphohistiocytosis or macrophage activation syndrome (sHLH/MAS) due to its potential mortality benefits and possible steroid-sparing effects.^2^ For these indications, the induction dose of IV anakinra is recommended to be at least 1 to 2 mg/kg per day (max 8 mg/kg per day) followed by the fixed maintenance dose of 100 mg daily and subsequent conversion to subQ anakinra.^2^ The higher doses are often required in critically ill pediatric patients. The highest dose for HLH given to a patient in our study was 10 mg/kg/day.

Anakinra has established efficacy in systemic juvenile idiopathic arthritis related MAS, but the optimal IV dosing regimen remains unknown.^2^ A retrospective case series reported a 4-year-old female with systemic juvenile idiopathic arthritis receiving IV anakinra at a dose of 5.6 mg/kg every 12 hours for an unknown duration, followed by maintenance SubQ anakinra 0.9 mg/kg/day with a follow-up of 24 months.^2,19^ In a more recent study, 10 pediatric patients aged from 1 to 20 years with SoJIA received IV anakinra at the initial dose of 1.7 to 20 mg/kg/day and at the max dose of 6 to 20 mg/kg/day for a total treatment duration of 3 to 12 days.^20^ Similarly, the 2 patients with SoJIA flares in our study started IV anakinra 4 mg/kg/dose q24h and 2.5 mg/kg/dose q6h respectively, and 1 patient was transitioned to SubQ anakinra 1 week later.

The most common adverse drug reactions reported in the prescribing information for anakinra are mild-moderate injection site reactions (bruising, erythema, and pain), headache, nausea and vomiting, and arthralgia.^6,7^ However, none of these side effects were documented for the patients in this study. There have also been anecdotal reports of tachyphylaxis to anakinra as well as severe disease rebound with abrupt drug continuation in patients with SoJIA.^21^ Although one study patient with SoJIA developed tachyphylaxis after treatment with SubQ anakinra 7 years ago prior to admission, re-initiation of IV anakinra appeared to be effective for this SoJIA flare. No opportunistic infections were observed in the study patients. The patients that had concomitant EBV viremia and candidemia were diagnosed with these infections prior to initiation of IV anakinra.

Our study provides additional evidence for off-label indications of IV anakinra, corresponding dosing strategies, and relevant safety information, which supports its use for some cytokine storm syndromes in pediatric patients. Amid the COVID-19 pandemic, this retrospective study describes pertinent clinical characteristics of patients with cytokine storm syndromes and reviews the dosing regimens and safety profile of IV anakinra for treatment of MIS-C associated with COVID-19 to help inform future clinical practice. Although efficacy was not the major study endpoint, change in inflammatory biomarkers and the 30-day survival rate were collected for informational purposes.

Our study also has several limitations. First, correlation between the use of IV anakinra and the reduction in inflammatory biomarkers could not be confirmed because no comparison group was included. It is possible the reduction in these markers could have been the result of other immunomodulator use. Second, this study had a small sample size with high heterogeneity, making it difficult to establish the optimal dosing regimens of IV anakinra. Comparison between different dosing strategies of IV anakinra was also not conducted in this study. Third, the ability to detect adverse effects from a small retrospective study is limited. In addition, 3 obese patients received weight-based dosing of IV anakinra using actual body weight. In a pharmacokinetic (PK) study conducted in obese adult patients, the absolute bioavailability with IV anakinra given as a fixed dose of 100 mg ranged from 80% to 92% and was unrelated to BW or BMI; however, the change in pharmacokinetics of IV anakinra in obese pediatric patients remains unknown.^22^ Given the wide therapeutic window of anakinra, the possible PK differences were less likely to impact the effectiveness of IV anakinra.

Overall, because of the retrospective nature of this single-center cohort study, confounders that may bias study results cannot be precluded, such as prior use of immunomodulators like IVIG and corticosteroids. Current recommendations for the off-label use of IV anakinra are mainly based on lower quality evidence such as case reports. Multicenter compilation of MIS-C data is needed to further elucidate appropriate treatment for these patient populations. Robust PK studies specifically in pediatric patients with renal dysfunction and critical illness could be considered in the future. Ideally, randomized controlled clinical trials should be designed to evaluate the safety and efficacy of IV anakinra for treatment of pediatric cytokine storm syndromes.

## Conclusion

Intravenous anakinra therapy was used off-label for treatment of MIS-C associated with COVID-19, HLH, and management of SoJIA flares in critically ill pediatric patients including those with previous glucocorticoid or IVIG treatment at an academic medical center with no adverse drug events reported. This medication use evaluation of IV anakinra provides additional information regarding its dosing and use in pediatric patients. Clinicians should consider individual patient characteristics and previous response to immunomodulatory therapies before initiating IV anakinra. Future larger studies are needed to confirm efficacy and safety of off-label use of IV anakinra.

## Supplemental Material

sj-docx-1-hpx-10.1177_00185787221142470 – Supplemental material for Use of Intravenous Anakinra for Management of Pediatric Cytokine Storm Syndromes at an Academic Medical Center Click here for additional data file. Supplemental material, sj-docx-1-hpx-10.1177_00185787221142470 for Use of Intravenous Anakinra for Management of Pediatric Cytokine Storm Syndromes at an Academic Medical Center by Lirong Yang, Sandra Lowry and Travis Heath in Hospital Pharmacy
