# Supplementary material for: Use of Intravenous Anakinra for Management of Pediatric Cytokine Storm Syndromes at an Academic Medical Center
Source: Hosp Pharm. 2023 Feb 14;58(4):376–81. doi: 10.1177/00185787221142470 (PMC10288450; doi:10.1177/00185787221142470)
Supplement: sj-docx-1-hpx-10.1177_00185787221142470 – Supplemental material for Use of Intravenous Anakinra for Management of Pediatric Cytokine Storm Syndromes at an Academic Medical Center [file sj-docx-1-hpx-10.1177_00185787221142470.docx]

**Appendix-**Intravenous (IV) Anakinra Dosing Regimen for Primary Indications

| Age - years | Weight - kg | Dosing Regimen | Treatment Duration - days |
| --- | --- | --- | --- |
| MIS – C Associated with COVID-19 Infection | | |  |
| 7 | 35 | 2 mg/kg IV q12h for 6 days followed by 2 mg/kg IV q24h for 3 days | 9 |
| 4 | 17 | 2 mg/kg IV q12h for 2 days followed by 1 mg/kg IV q12h for 2 days | 4 |
| 4 | 19 | 2 mg/kg IV q12h | 5 |
| 5 | 14 | 4 mg/kg IV q12h for 5 days followed by 2 mg/kg IV q12h for 2 days | 6 |
| 8 | 19 | 5 mg/kg IV q12h for 3 days followed by 3.5 mg/kg IV q12h for 8 days | 10 |
| 11 | 82 | 2.5 mg/kg IV q12h for 2 days followed by 2.5 mg/kg IV q6h for 4 days and then 2.5 mg/kg IV q12h for 2 days | 7 |
| 3 | 27 | 4 mg/kg IV q8h for 1 day followed by 4 mg/kg IV q6h for 2 days, transition to anakinra 4 mg/kg subcutaneously q6h for 7 days, then switch to 4 mg/kg IV q12h for 6 days followed by 4 mg/kg and 2 mg/kg IV q24h for 4 days and wean down to 4 mg/kg IV q24h for 4 days | 20 |
| 16 | 64 | 2 mg/kg IV q12h for 4 days followed by 4 mg/kg IV q12h for 8 days and wean down to 4 mg/kg IV q24h for 2 days | 13 |
| HLH | | |  |
| 21 | 81 | 1.25 mg/kg IV q6h | 1 |
| 5 | 14 | 10 mg/kg IV q24h | 14 |
| 12 | 26 | 5 mg/kg IV q24h | 11 |
| SoJIA flares | | |  |
| 1.8 | 9 | 4 mg/kg IV q24h | 6 |
| 9 | 38 | 2.5 mg/kg IV q6h for 3 days followed by 2.5 mg/kg IV q8h for 2 days and wean down to 2.5 mg/kg IV q12h for 1 day, then transition to 2.5 mg/kg subcutaneously q12h for 2 days | 7 |
| Possible cytokine storm/hyperinflammatory state | | |  |
| 0.75 | 7 | 2 mg/kg IV once followed by 4 mg/kg IV q12h for 2 days | 3 |

Abbreviations: COVID-19, coronavirus disease 19; HLH, hemophagocytic lymphohistiocytosis; MIS-C, multisystem inflammatory syndrome in children; SoJIA, Systemic onset juvenile idiopathic arthritis
